# Validation of the English version of the arrhythmia-specific questionnaire in tachycardia and arrhythmia (ASTA): a Rasch evaluation study

**DOI:** 10.1186/s41687-022-00493-4

**Published:** 2022-08-26

**Authors:** Ulla Walfridsson, Håkan Walfridsson, Melissa E. Middeldorp, Prashanthan Sanders, Kristofer Årestedt

**Affiliations:** 1 Department of Medical and Health Sciences, Linköping University, Linköping, Sweden; 2 Department of Cardiology, University Hospital, Linköping, Sweden; 3 Centre for Heart Rhythm Disorders, University of Adelaide, South Australian Health & Medical Research Institute and Royal Adelaide Hospital, Adelaide, Australia; 4 Faculty of Health and Life Sciences, Linnaeus University, Kalmar, Sweden; 5 The Research Section, Region Kalmar County, Kalmar, Sweden

**Keywords:** Arrhythmias, Atrial fibrillation, Health-related quality of life, Symptom burden, Rasch analysis, Validation

## Abstract

**Background:**

Patient-reported outcome measures are important in person-centered care, providing valuable information about patients’ experiences. Disease-specific questionnaires add important information about a certain disease in comparison to generic questionnaires. Questionnaires need to be validated in the targeted population to achieve reliable data. The purpose with the study was to use Rasch measurement theory to evaluate the English version of the ASTA questionnaire.

**Methods:**

The Rasch model theory was used to evaluate global and item fit, targeting, response category functioning, local independency, unidimensionality, differential item functioning (DIF) for gender and age, and reliability.

**Results:**

The study included 202 patients undergoing DC conversion or catheter ablation at the Centre for Heart Rhythm Disorders at the University of Adelaide, Australia. The mean age was 67 years and 30% were women. Most patients had atrial fibrillation (n = 179), others had atrial flutter or had a combination. One of nine items demonstrated unsatisfactory model fit in the ASTA Symptom scale and two of 13 in the ASTA Health-Related Quality of Life (HRQoL) scale. Unidimensionality was supported for both scales. The targeting was acceptable except for the lower end of the scales. Both scales showed reversed thresholds for the response categories “quite a lot” and “a lot” (eight of ASTA symptoms and 12 of ASTA HRQoL items). Some problems with local dependency were detected in both scales. The reliability (person separation index) was satisfactory: 0.75 for the ASTA symptom scale and 0.77 for the ASTA HRQoL scale. No DIF for gender and age were detected.

**Conclusions:**

The English version of the ASTA questionnaire demonstrated satisfactory measurement properties according to the Rasch model. However, it needs to be evaluated in patients with other arrhythmias. The response categories should be considered as well as DIF in further validation. The ASTA questionnaire can be used for assessments of symptoms and HRQoL between groups of different ages and genders in patients with arrhythmia.

## Introduction

Atrial fibrillation (AF), the most common arrhythmia, is known to cause a pronounced symptom burden and negative impact on health-related quality of life (HRQoL) [1].

Treatment of AF is primarily driven by symptoms and therefore needs to be evaluated in clinical care. The European Society of Cardiology (ESC) guidelines (2020) specifically highlight the importance of assessments with patient-reported outcome measures (PROMs) [1]. Patient-reported outcome measures are important in person-centered care, providing valuable information about patients’ experiences. Disease-specific questionnaires give important information about symptoms and consequences on daily life concerns related to a certain disease in comparison to generic questionnaires. To achieve reliable data, questionnaires need to be validated in the targeted population [2].

Several PROMs have been developed to assess symptom burden and HRQoL in patients with arrhythmia, specifically in patients with AF. A scoping review showed that the Arrhythmia-Specific questionnaire in Tachycardia and Arrhythmia (ASTA) was one of the measures that covered most functions according to the International Classification of Functioning (ICF) [3]. The ASTA questionnaire was developed and validated in Sweden among patients with different forms of arrhythmias [4, 5]. The original Swedish version has been evaluated regarding content validity, construct with convergent and discriminant validity, factor structure item-total correlations and internal consistency alongside the Short Form 36 (SF-36) and Symptom Checklist Frequency and Severity scale (SCL). The questionnaire has been translated into several languages, including Danish, Polish, Brazilian Portuguese, and German, and into an English version [6, 7]. However, even if the ASTA questionnaire was translated into English some years ago, the English version has not yet been validated. The ASTA questionnaire has earlier only been evaluated by classical test theory (CTT) methods. One limitation with CTT is that statistics used to describe the item parameters are sample dependent. This implies that the measurement properties may vary across different groups and samples [8]. In contrast, item response theory, including Rasch measurement theory, makes strong assumptions on monotonicity, unidimensionality, local independence, and invariance. In addition, item parameters produced by item response theory, including Rasch measurement theory, are invariant across different samples, i.e., these statistics are not sample dependent [8, 9]. Thus, Rasch measurement theory is commonly recommended to evaluate self-reported measures [8].

## Methods

### Aim

The aim of this study was to use Rasch measurement theory to evaluate the English version of the ASTA questionnaire.

### Description of the stages in the study process

This psychometric evaluation study was conducted in two stages: I) the earlier performed translation process of the ASTA questionnaire and II) the evaluation of the measurement properties.

### Step I: the translation process of the ASTA questionnaire

#### The arrhythmia-specific questionnaire in tachycardia and arrhythmia (ASTA) questionnaire design

The ASTA questionnaire is aimed to evaluate symptoms and HRQoL in patients with different forms of arrhythmias such as AF, atrial flutter (AFL), Wolff-Parkinson-white syndrome, AV-nodal reentrant tachycardia and those with ventricular arrhythmia and premature ventricular extra beats, and is divided into three parts. Part I evaluates the last episode of arrhythmia, current medication, and the presence of arrhythmia at the time of follow up. Part II measure arrhythmia-specific symptoms, the ASTA nine-item symptom scale. The response format is a four-point Likert type scale; “No” (0); “Yes, to a certain extent” (1); “Yes, quite a lot” (2) and “Yes, a lot” (3). The responses can be summarized and transformed to a scale score ranging between 0 and 100 (raw score; lowest possible score/possible score range × 100), where a higher score implies higher symptom burden due to the arrhythmia. Outside of the symptom scale, there is one item concerning frequency, two about duration, and one about palpitations. The patients are asked if there are factors influencing arrhythmia occurrence and the experience of near syncope and/or syncope in connection with arrhythmia. Part III measures the arrhythmia’s influence on daily life concerns with the ASTA 13-item HRQoL scale, which has the same response alternatives as the ASTA symptom scale. Using the same scoring as for the ASTA symptom scale, a total score can be calculated, where higher scores reflect a worse effect on HRQoL. In additional, two subscales can be used, a physical including seven items, and a mental including six items. (Supplement 1) This study focused on evaluating the ASTA symptom burden and HRQoL scales with Rasch analysis. Therefore, both were treated as unidimensional scales in the present study.

#### The translation process

The Swedish version has been translated into English some years ago using well-recognized techniques inspired by The Professional Society for Health Economics and Outcomes Research (ISPOR), with translations by native English and Swedish speaking persons [10].

The ASTA questionnaire was initially translated into English by a native Swedish-speaking person who had lived in Great Britain for ten years. The version was discussed in the research team, which included the constructors of the ASTA questionnaire. Thereafter the English version was evaluated by a Swedish electrophysiologist who had worked in Canada and who was skilled in the treatment of patients with arrhythmias. In the next step, the two versions were examined by a translation agency. Finally, the English version was discussed in a focus group with four healthcare professionals. Three of them were native Swedish-speaking physicians, skilled in English and working with patients with arrhythmias. The person who originally translated the ASTA questionnaire confirmed the result, with one correction for wording (one item in the HRQoL scale). The process continued with two native English-speaking persons, not involved in the translation process, filled out the English version of the ASTA questionnaire. As a part of the validation work the patients at the clinic in Adelaide, Australia were asked to consider the wording and to comment on whether or not there were any uncertainties**.**

### Step II: evaluation of the measurement properties

#### Study population

The study involved patients from the Centre for Heart Rhythm Disorders at the University of Adelaide, Australia. Symptomatic patients who were referred for treatment of AF and/or AFL including ablation and cardioversion during November 2017 until February 2019 were approached to take part in the study by completing the ASTA questionnaire. Patients were included if they were ≥ 18 years, symptomatic, proficient in English and physically and mentally able to complete the questionnaire. In total, 212 patients were invited to participate, of which 205 agreed to complete the ASTA questionnaire. Of the returned questionnaires (n = 205), three were incomplete and were excluded, so the final sample included 202 patients.

#### Completion of the questionnaire

Patients were asked to complete the questionnaire prior to their appointment with their cardiologist and before treatment. This was done so the patient was not informed of their rhythm prior to completing the form, in order to avoid influencing their responses. The first 26 patients filled out the ASTA questionnaire in its paper version and the others via its web-version.

**Fig. 1:**
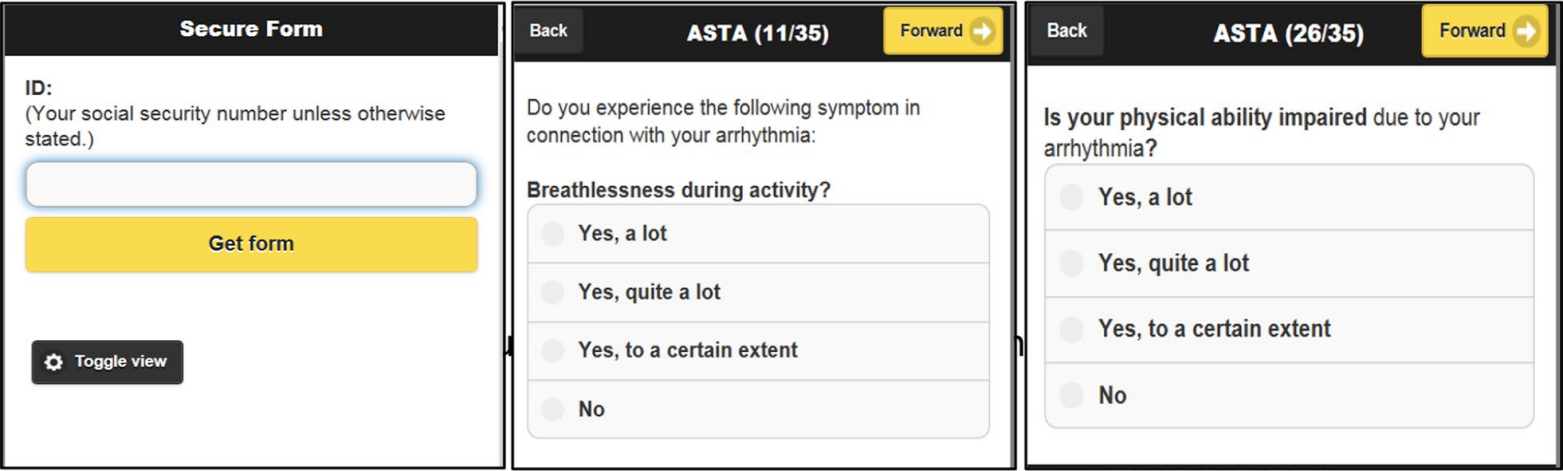
ASTA website interface. ASTA=Arrhythmia-Specific questionnaire in Tachycardia and Arrhythmia

The questionnaire was made available via a website interface (Fig. 1). Patients would log onto the web-based interface and enter a unique identification number, then proceed through the questionnaire. The interface, provided by Nordsoft AB Sweden, enabled easy use with single click answer selection. On completion, the questionnaire was uploaded to a secure internet-based database for storage and analysis.

As a part of validation of the English version of the ASTA questionnaire the patients were asked to consider the wording and to comment on whether or not there were any uncertainties**,** but no one pointed anything out concerning this. The most common issue the patients encountered concerned medications. For some, questions regarding frequency and duration of AF episodes rendered in some perplexities.

### Statistical analysis

Descriptive statistics were used to present the study population and to evaluate data quality in terms of score distributions for items and scales. Floor and ceiling effects are commonly defined if more than 20% of the respondents achieve the lowest and/or highest scores [11], which was adopted in the present study. These data were analyzed using Stata version 16.1 (StataCorp LP, College Station, TX).

The unidimensional Rasch model for ordered categories (unrestricted polytomous Rasch model) was used to evaluate the ASTA symptom and HRQoL scales [12]. The Rasch analysis was undertaken using RUMM2030 version 5.4 (Rumm Laboratory Pty Ltd, Duncraig, Australia). The analyses were based on five class intervals (i.e., persons with similar levels on the ASTA symptom and HRQoL scales respectively) to ensure a sufficiently large number of persons in each (n ≥ 30). Nine patients had extreme scores (i.e., reported the highest or lowest possible scores on all items) on the ASTA symptom scale and 20 on the ASTA HRQoL scale. In the Rasch model, extreme scores correspond to infinite or indefinite measures on the latent variable and are therefore not estimable. In RUMM, persons with extreme scores are therefore provided with a tentative estimate of their location parameter [12].

The following aspects were evaluated:

*Global model fit* A perfect global model fit is reflected by mean residual values close to 0 and standard deviations close to 1 for both item and persons. Moreover, the total item trait interaction (chi-square based statistics) should be non-significant [13].

*Individual item fit* Individual fit of items is reflected by standardized fit residual values within the range ± 2.5 and non-significant Bonferroni corrected p-values [11]. The Bonferroni corrected p-value depends on the number of items and was therefore set at p < 0.006 for the ASTA symptom scale and p < 0.004 for the ASTA HRQoL scale. The individual item fit was also graphically examined using the item characteristic curves. These curves illustrate the probability of a correct response dependent on the person’s ability (level on the latent variable) and the item difficulty [12].

*Response categories functioning* The ordering of the centralized thresholds for each item was inspected to evaluate the response categories functioning. Thresholds can be defined as the point between two response categories where either response is equally probable. Therefore, disorder thresholds may indicate that the scoring function (i.e., response categories) is not working as intended [11].

*Local independency* An important assumption of the Rasch model is that items in a test should not be related to each other after the effect of the latent variable is conditioned out. Violations to this assumption, i.e., local dependency, are reflected by high correlations between item residuals. Different critical values have been suggested but correlations greater than 0.2 above the average of all item residual correlations have been suggested as problematic in most situations [14].

*Unidimensionality* Another important assumption of the Rasch model is that the latent variable is unidimensional, i.e., that all items reflect one underlying construct. This assumption is commonly confirmed by satisfactory model fit statistics and lack of response dependency (i.e., local independency) [15]. A combined principal component analysis (PCA) of residuals and t-test approach was used in the present study. Items with the strongest positive and negative loadings on the first principal component were used to estimate separate person locations and associated standard errors. A series of t-tests was then conducted to compare person locations based on the two different subsets of items. Fewer than 5% of the t-tests are supposed to be significant (p < 0.05), alternatively the lower bound of the Agresi-Coull binominal 95% confidence interval should overlap by 5% to support unidimensionality [16, 17].

*Person-item threshold distribution* This aspect reflects to what extent the item difficulty represents person ability, i.e., level of symptoms and HRQoL among the respondents. For this purpose, the item thresholds were compared with the person ability level. The mean person location is expected to be around the mean item threshold location, i.e., 0 logits. In addition, the item thresholds are expected to cover about the same range of the logit scale as person locations [11].

*Person separation index* The person separation index reflects the ability of the measure to discriminate between persons with different levels of the construct. It is also a measure of internal consistency, analogous to Cronbach’s alpha. Thus, the person separation index is expected to exceed 0.7 to support reliability [11]. In the present study, also ordinal alpha and Cronbach’s alpha were calculated to examine internal consistency [18, 19].

*Differential item functioning for age and gender* Differential item functioning (DIF) implies that different groups have comparable levels of the latent variable but respond differently to individual items. To detect DIF for age and gender, a two-way analysis of variance across these person factors and class intervals was conducted [13] Age was classified as younger (< 65 years) and older (≥ 65 years). The main effect of the person factors was used to detect uniform DIF while the interaction effect between the person factor and class intervals was used to detect non-uniform DIF. Due to the large number of comparisons, Bonferroni corrections were applied: p < 0.002 was used to detect DIF for the ASTA symptom scale and p < 0.001 for the ASTA HRQoL scale.

## Results

### Patient characteristics

The final sample included 202 patients: 61 (30%) women and 141 (70%) men. The mean age was 67 years (SD = 12.2). Two thirds of the patients (n = 128) were treated with catheter ablation and one third (n = 72) with DC conversion. Most patients had AF (n = 179) while a few had AFL (n = 3) or a combination of AF and AFL (n = 18). The most common anti-arrhythmic medications were beta blockers (n = 79), followed by class III (n = 62), and Ca-channel blockers (n = 41). Most patients (n = 187) were on anti-coagulants, i.e., New Oral Anti-Coagulants (NOACs), Warfarin or thrombocyte inhibitor, where NOACs were the most commonly used (n = 150). Other common medications were statins and AII-inhibitors (Table 1).

**Table 1:** Patient characteristics (n = 202)

Age (years), mean (SD) [range]	66.9 (12.2) [20–94]
Gender, n (%)	
Women	61 (30.2)
Men	141 (69.8)
Type of atrial arrhythmia, n (%)	
Arial fibrillation	179 (88.6)
Atrial flutter	3 (1.5)
Combination of atrial fibrillation and atrial flutter	18 (8.9)
Missing data	2 (0.9)
Treatment, n (%)	
DC-conversion	72 (35.6)
Catheter ablation	128 (63.3)
Missing data	2 (0.9)
Medication, n (%)	
Anti-coagulants	187 (92.5)
NOAC	150 (74.3)
Warfarin	21 (10.4)
Thrombocyte inhibitor	16 (7.9)
Anti-arrhythmics, n (%)	
Beta blockers	79 (39.6)
Ca-channel blockers	41 (20.3)
Digoxin	6 (3.0)
Class Ic	24 (11.9)
Class III	62 (30.7)
Other medications, n (%)	
Statins	76 (37.6)
ACE-inhibitors	54 (26.7)
AII-inhibitors	74 (36.6)

*AF* Atrial fibrillation,* AFL* Atrial flutter,* ASTA* Arrhythmia-Specific questionnaire in Tachycardia and Arrhythmia,* CFA* Confirmatory Factor Analysis,* CTT* Classic Test Theory,* DIF* Differential Item Functioning,* ESC* European Society of Cardiology,* HRQoL* Health-Related Quality of Life,* NOACs* New Oral Anti-Coagulants,* ICF* International Classification of Functioning,* ISPOR* Professional Society for Health Economics and Outcomes Research,* PROMs* Patient-Reported Outcome Measures,* PCA* Principal Component Analysis,* SCL* Symptom Checklist Frequency and Severity scale

### Data quality

The amount of missing data was low: three missing values in ASTA symptom scale and two items in the ASTA HRQoL scale. Floor effects were shown for all items in the ASTA symptom scale and the ASTA HRQoL scale. Ceiling effects were rare, only shown in one item about breathlessness during activity (item 1) in the ASTA symptom scale (Table 2).

**Table 2:** Item statistics of the ASTA Symptom scale and ASTA Health-Related Quality-of-Life scale

ASTA scales and items	Mdn (Q1;Q3)	Item score distribution, n (%)				
		No (0)	Yes, to a certain extent (1)	Yes, quite a lot (2)	Yes, a lot (3)	Missing
*ASTA Symptom scale (n = 200)*						
1 Breathlessness during activity	1 (0;2)	59 (29.5)	64 (32.0)	29 (14.5)	46 (23.0)	2 (1.0)
2 Breathlessness even at rest	0 (0;1)	118 (59.0)	62 (31.0)	7 (3.5)	12 (6.0)	1 (0.5)
3 Dizziness	1 (0;1)	93 (46.5)	79 (39.5)	17 (8.5)	11 (5.5)	–
4 Cold sweats	0 (0;1)	149 (74.5)	35 (17.5)	10 (5.0)	6 (3.0)	–
5 Weakness/fatigue	1 (0;2)	58 (29.0)	77 (38.5)	31 (15.5)	34 (17.0	–
6 Tiredness	1 (0;2)	52 (26.0)	69 (34.5)	40 (20.0)	39 (19.5)	–
7 Chest pain	0 (0;0)	153 (76.5)	41 (20.5)	4 (2.0)	2 (1.0)	–
8 Pressure/discomfort in chest	0 (0;1)	125 (62.5)	61 (30.5)	9 (4.5)	5 (2.5)	–
9 Worry/anxiety	1 (0;1)	68 (34.0)	95 (47.5)	18 (9.0)	19 (9.5)	–
*ASTA Health-Related Quality-of-Life scale (n = 202)*						
1 Feel unable to work, study or carry out daily activities	0 (0;1)	103 (51.0)	62 (30.7)	19 (9.4)	18 (8.9)	–
2 Spend less time with family/relatives and friends	0 (0;0)	157 (77.7)	32 (15.8)	9 (4.5)	4 (2.0)	–
3 Spend less time with acquaintances	0 (0;0)	152 (75.3)	39 (19.3)	7 (3.5)	4 (2.0)	–
4 Avoid planning things you would like to do	0 (0;1)	116 (57.4)	54 (26.7)	13 (6.4)	19 (9.4)	–
5 Impaired physical ability	1 (0;1)	78 (38.6)	74 (36.6)	27 (13.4)	23 (11.4)	–
6 Impaired ability to concentrate	0 (0;1)	137 (67.8)	48 (23.8)	12 (5.9)	5 (2.5)	–
7 Feel dejected or sad	0 (0; 1)	120 (59.4)	57 (28.2)	16 (7.9)	9 (4.5)	–
8 Feel irritated or angry	0 (0;1)	128 (63.4)	54 (26.7)	11 (5.5)	9 (4.5)	–
9 Experience sleep problems	0 (0;1)	112 (55.5)	66 (32.7)	19 (9.4)	5 (2.5)	–
10 Negatively affected sexual life	0 (0;1)	144 (71.3)	33 (16.3)	9 (4.5)	15 (7.4)	1 (0.5)
11 Afraid of dying	0 (0;1)	127 (62.9)	63 (31.2)	8 (4.0)	4 (2.0)	–
12 Deteriorated life situation	1 (0;1)	100 (49.5)	70 (34.7)	17 (8.4)	14 (6.9)	1 (0.5)
13 Feel worried that symptoms will re-occur during arrhythmia-free periods	1 (0;1)	88 (43.6)	84 (41.6)	17 (8.4)	13 (6.4)	–

*ASTA* Arrhythmia-Specific questionnaire in Tachycardia and Arrhythmia

### Rasch analysis

#### Global model fit

The mean residual values for the items deviated only slightly from 0 in the ASTA symptom scale (− 0.29) and the ASTA HRQoL scale (− 0.30) while the standard deviation exceeded the optimal value of 1 for both scales (1.76 and 1.88 respectively). The mean residual values for persons also deviated slightly from 0 in both scales (− 0.26 and − 0.27 respectively) while the standard deviation for the fit residuals was close to 1 (0.89 and 1.02 respectively). The total item trait interaction statistics indicated misfit to the Rasch model for both the ASTA symptom scale [χ^2^(36) = 63.25, *p* = 0.003] and the ASTA HRQoL scale [χ^2^(52) = 110.73, *p* < 0.001] (Table 3).

**Table 3:** Global fit statistics and reliability for the ASTA Symptom scale and ASTA Heath-Related Quality of Life scale

	ASTA symptom scale	ASTA HRQoL scale
Items		
Location, mean	0.00	0.00
Location, SD	0.82	0.45
Fit residual, mean	− 0.29	− 0.30
Fit residual, SD	1.76	1.88
Persons		
Location, mean	− 1.16	− 1.60
Location, SD	1.19	1.31
Fit residual, mean	− 0.26	− 0.27
Fit residual, SD	0.89	1.02
Total item trait interaction		
Total item χ^2^	63.25	110.73
df	36	52
*p* value	0.003	< 0.001
Person separation index	0.75	0.77
Ordinal alpha	0.86	0.92
Cronbach’s alpha	0.81	0.88

*ASTA* Arrhythmia-Specific questionnaire in Tachycardia and Arrhythmia, *HRQoL* Health-Related Quality of Life

#### Individual item fit

The item about worry/anxiety (item 9) in the ASTA symptom scale had standardized fit residual values above 2.5 (2.85). The item characteristic curve showed that persons with low symptom levels tended to score higher than expected while persons with high symptom levels tended to score lower than expected on this item according to the Rasch model. However, the Bonferroni corrected p-value was non-significant (*p* = 0.046). In the ASTA HRQoL scale, two items had standardized fit residual values exceeding ± 2.5 (2.90 and − 2.87). Examination of the item characteristic curves showed that the item about sleep (item 9) had the same problem as item 9 in the ASTA symptom burden scale. In contrast, the item characteristic curve for the item about deteriorated life situation (item 12) showed the opposite problem, i.e., that persons with low HRQoL tended to score lower than expected while persons with high HRQoL tended to score higher than expected on this item according to the Rasch model. However, the Bonferroni corrected p-values were non-significant for both items (*p* = 0.051 and p = 0.020) (Table 4).

**Table 4:** Item location, item fit statistics and thresholds for the items in the ASTA Symptom scale and ASTA Health-Related Quality of Life scale

Items^a^	Item location	Item fit statistics		Centralized thresholds			
		Residual^b^	*p* value^c^	I	II	III	Reversed
*ASTA Symptom Scale*							
6	− 0.95	− 2.27	0.008	− 1.18	0.54	0.63	No
1	− 0.92	− 0.75	0.599	− 0.86	0.92	− 0.06	Yes
5	− 0.77	− 2.36	0.244	− 1.16	0.81	0.35	Yes
9	− 0.39	**2.85**	0.046	− 1.34	0.37	− 0.03	Yes
3	0.06	1.39	0.522	− 1.18	0.91	0.27	Yes
2	0.31	− 1.49	0.055	− 0.86	0.56	− 0.69	Yes
4	0.57	0.86	0.194	− 0.06	0.34	− 0.27	Yes
8	0.69	0.28	0.447	− 1.13	0.88	0.24	Yes
7	1.40	− 1.13	0.391	− 1.04	0.62	0.42	Yes
*ASTA HRQoL Scale*							
5	− 0.83	− 2.08	0.007	− 1.36	0.91	0.45	Yes
1	− 0.42	− 2.41	0.152	− 0.86	0.68	0.18	Yes
13	− 0.40	1.90	0.125	− 1.31	0.93	0.23	Yes
4	− 0.35	1.23	0.012	− 0.53	0.05	− 0.52	Yes
12	− 0.31	**− 2.87**	0.020	− 1.16	0.93	0.23	Yes
10	− 0.06	0.56	0.399	0.10	0.73	− 0.83	Yes
7	0.02	0.08	0.679	− 0.88	0.56	0.32	Yes
8	0.10	0.25	0.563	− 0.76	0.80	− 0.04	Yes
9	0.18	**2.90**	0.051	− 1.21	0.26	0.95	No
11	0.36	1.45	0.024	− 1.09	0.02	0.07	Yes
6	0.38	− 0.73	0.391	− 0.79	0.46	0.32	Yes
3	0.63	− 1.83	0.186	− 0.68	0.85	− 0.17	Yes
2	0.69	− 2.30	0.105	− 0.48	0.34	0.14	Yes

*ASTA* Arrhythmia-Specific questionnaire in Tachycardia and Arrhythmia, *HRQoL* Health-Related Quality of Life

^a^Items are sorted in location order, from the easiest to the most difficult

^b^Residuals ± 2.5 are marked in bold

^c^The Bonferroni corrected *p* values are *p* < 0.006 for the ASTA Symptom scale and *p* < 0.004 for the ASTA HRQoL scale

### Response categories functioning

**Fig. 2.**
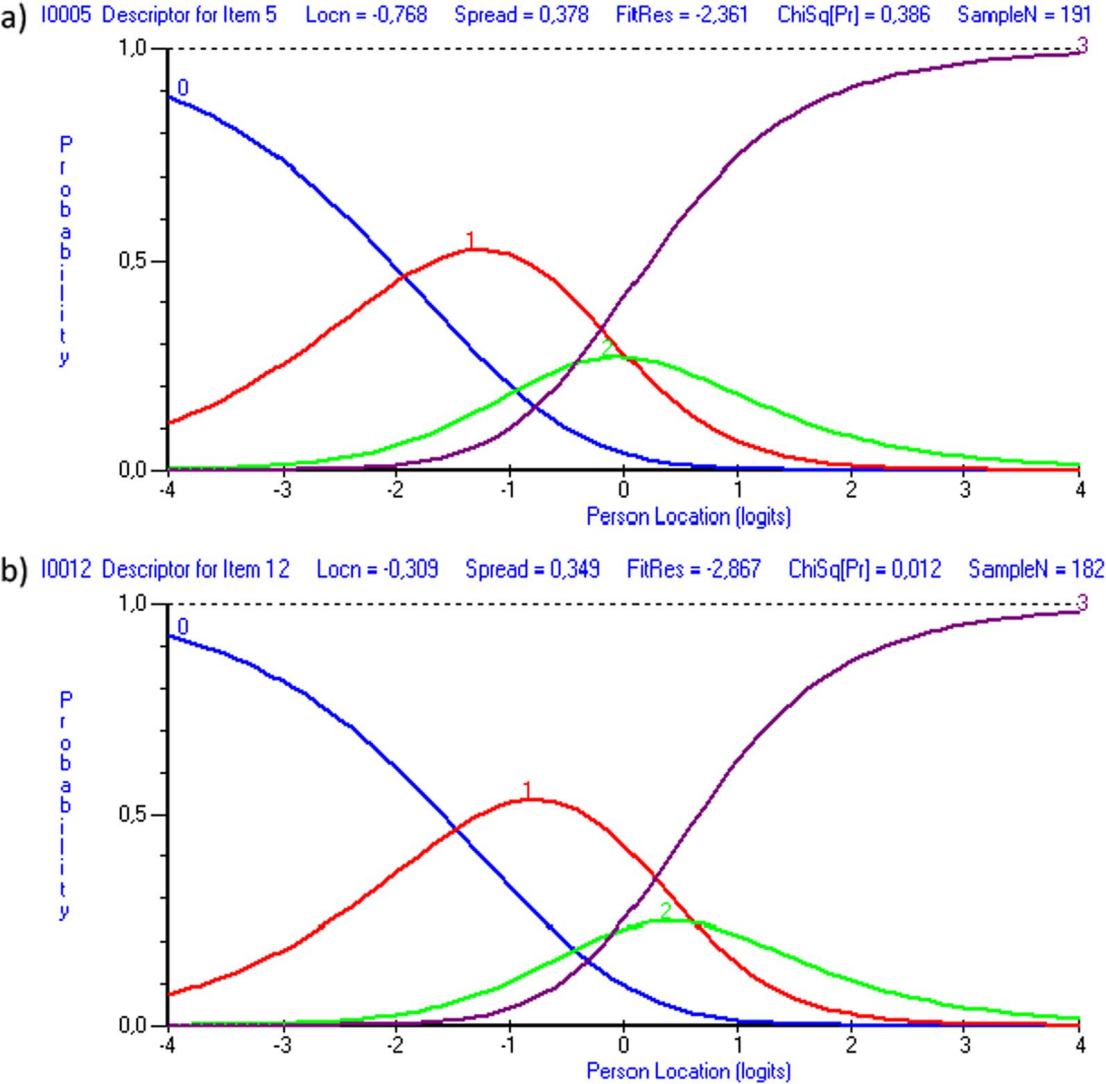
Examples of category probability curves that illustrate the problem with disordered thresholds. **a** Illustrates this problem for item 5 in the Arrhythmia-Specific questionnaire in Tachycardia and Arrhythmia (ASTA) Symptom scale and **b** illustrates this problem for item 12 in the ASTA Health-Related Quality of Life scale. As can be seen in both illustrations, persons tend to use the highest response category (curve 3) before the next highest response category (curve 2)

Disordered thresholds were found in all items except the item about tiredness (item 6) in the ASTA symptom scale and all items except the item about sleep (item 9) in the ASTA HRQoL scale (Table 4). These problems were all related to thresholds two and three. As an example, Fig. 2a illustrates disordered thresholds for item about weakness/fatigue (item 5) in the ASTA symptom scale and Fig. 2b illustrates the same problem for the item about deteriorated life situation (item 12) in the ASTA HRQoL scale.

#### Local independency

Some problems with local dependency were detected in both scales. The mean residual item correlation in the ASTA symptom scale was -0.11, which implies that values above 0.09 were considered as problematic. According to this, problems were detected between the following item pairs: 7 and 8 (0.41, *chest pain* vs. *pressure/discomfort in the chest*), 5 and 6 (0.40, *weakness/fatigue* vs. *tiredness*), 1 and 2 (*breathlessness during activity* vs. *breathlessness even at rest*). The mean residual correlation in the ASTA HRQoL scale was 0.08, which implies that correlations above 0.12 were considered as problematic. According to this, problems were detected between the following item pairs: 2 and 3 (0.59, *spend less time with family/relatives and friends* vs. *spend less time with acquaintances*), 5 and 12 (0.29, *impaired physical ability* vs. *deteriorated life situation*), 1 and 5 (0.25, *feel unable to work, study or carry out daily activities* vs. *impaired physical ability*), 11 and 13 (0.17, *afraid of dying* vs. *feel worried that symptoms will re-occurring during arrhythmia-free periods*), 1 and 12 (0.14, *feel unable to work, study or carry out daily activities* vs. *deteriorated life situation*).

### Unidimensionality

Based on the PCA of residuals, the two subsets of items for the ASTA symptom scale included the following items: 1, 5, 6 vs. 4,7,8,9. More than 5% of the patients had significantly different scores on the two subsets of items (5.5%), but the lower bound of the binominal 95% CI overlapped 5% (0.03–0.10). The two subsets of items for the ASTA HRQoL scale included the following items: 1, 2, 4, 5, 12 vs. 7,8,9,11,13. Fewer than 5% of the patients had significantly different scores on the two subsets of items (4.6%) and the binominal 95% CI was 0.03–0.10. Thus, unidimensionality was supported for both scales based on the t-test approach.

#### Person-item threshold distribution

**Fig. 3.**
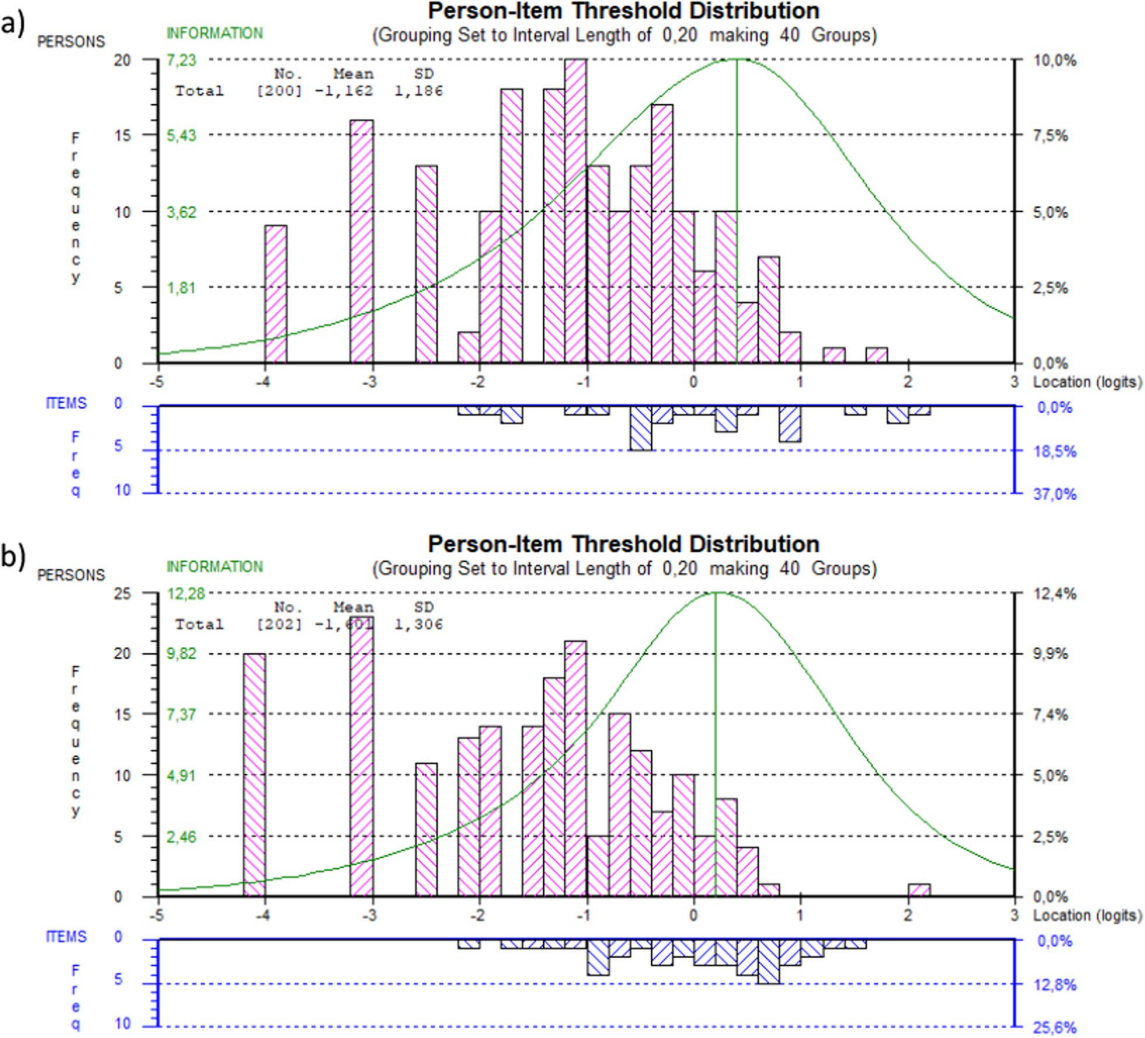
The person-item threshold distribution illustrates the targeting between person ability and item difficulty, i.e., the item parameters along the common logit scale. **a** Illustrates the distribution for the Arrhythmia-Specific questionnaire in Tachycardia and Arrhythmia (ASTA) Symptom scale and **b** Illustrates the distribution for the ASTA Health-Related Quality of Life scale. The item mean location is centralized at 0

The person-item threshold distribution for ASTA symptoms and HRQoL scales are shown in Fig. 3a, b. Both scales covered approximately ± 2 logits of the person ability scores. Persons with low symptom burden and higher HRQoL were not sufficiently covered by the item thresholds.

#### Person separation index and Cronbach’s alpha

The person separation index was satisfactory: 0.75 for the ASTA symptom scale and 0.77 for the ASTA HRQoL scale. The ordinal alpha values were 0.86 and 0.92 while the corresponding Cronbach’s alpha values were 0.81 and 0.88 respectively (Table 3).

### Differential item functioning (DIF)

No uniform or non-uniform DIF for age and gender were detected for the items in the ASTA symptom scale and ASTA HRQoL scale (Table 5).

**Table 5:** Differential item functioning for age and gender of the ASTA Symptom scale and ASTA Health-Related Quality of Life scale

Items	Differential item functioning for age^a^		Differential item functioning for gender^a^	
	Uniform *p* value	Non-uniform *p* value	Uniform *p* value	Non-uniform *p* value
*ASTA Symptom scale*				
1	0.017	0.041	0.342	0.456
2	0.691	0.697	0.005	0.059
3	0.248	0.066	0.364	0.081
4	0.054	0.541	0.826	0.232
5	0.087	0.046	0.854	0.297
6	0.015	0.040	0.427	0.701
7	0.714	0.688	0.062	0.012
8	0.583	0.795	0.291	0.007
9	0.011	0.045	0.907	0.004
*ASTA HRQoL scale*				
1	0.215	0.147	0.165	0.031
2	0.324	0.759	0.182	0.052
3	0.504	0.985	0.762	0.844
4	0.007	0.037	0.210	0.643
5	0.150	0.043	0.534	0.813
6	0.871	0.710	0.797	0.339
7	0.054	0.145	0.110	0.346
8	0.136	0.646	0.204	0.453
9	0.451	0.945	0.111	0.453
10	0.285	0.882	0.002	0.033
11	0.005	0.030	0.581	0.301
12	0.680	0.519	0.395	0.403
13	0.015	0.047	0.082	0.206

*ASTA* Arrhythmia-Specific questionnaire in Tachycardia and Arrhythmia, *HRQoL* Health-Related Quality of Life

^a^Based on a two-way ANOVA with Bonferroni corrected *p* values, *p* < 0.002 for the ASTA Symptom scale and the ASTA HRQoL scale

## Discussion

This is the first study that has evaluated the psychometric properties of the English version of the ASTA questionnaire, and the first based on Rasch measurement theory. The results support the use of the ASTA questionnaire as a unidimensional measure of symptom and HRQoL respectively. In addition, the ASTA questionnaire can be used to make invariant assessments of symptom distress and HRQoL between groups of different age and gender.

All except three items demonstrated acceptable model fit. One belonged to the ASTA symptom scale (*worry/anxiety*) and two to the ASTA HRQoL scale (*sleep problems and deteriorated life situation*). However, the findings for these items were unambiguous; the standardized fit residuals were only somewhat outside the range of ± 2.5 and no item demonstrated significant deviations from the Rasch model. Despite minor deviations, these findings may explain the global misfit, reflected by the item trait interaction statistics for both ASTA symptoms and HRQoL scales. Although these three items indicated misfit to the Rasch model, they are of great conceptual importance for symptom distress and HRQoL in this patient population since patients with AF often report problems with worry and anxiety, sleep problems and a stressful life situation [20–22].

Problems with reversed thresholds were identified for nearly all items in the ASTA symptom and HRQoL scales. Both scales use the same four-point response options and the problems were seen between thresholds two and three. Reversed thresholds may reflect problems with the response scale but there are other reasons such as dependence among underlying items and variations in the discrimination between adjacent categories. In addition, response categories seldom endorsed can create problems with reversed thresholds. Thus, reversed thresholds do not need to reflect the ordering of the item response categories. [23]. The highest scores (“Yes, quite a lot” and “Yes, a lot”) were less used and some problems with local dependency were identified, i.e., factors that can explain this problem. Thus, no strong conclusions can be drawn and the problem with reversed thresholds should be addressed in future validation studies of the ASTA questionnaire.

Some problems with local dependency were detected in both the ASTA symptom and HRQoL scales. Item redundancy, i.e., items measuring the same thing, is a common reason for local dependency [14]. This can probably explain the problems with items about *weakness/fatigue* and *tiredness*, *chest pain* and *pressure/discomfort in chest*, and *breathlessness during activity* and *breathlessness even at rest* in the ASTA symptom scale. This may also explain the problems with local dependency in the ASTA HRQoL scale in the items about *spending less time with family/relatives and friends* and *spending less time with acquaintances*, worries about *afraid of dying* and *worried that the symptoms will re-occur*, and also regarding *unable to carry out daily activities, impaired physical ability* and *deteriorated life situation*. However, the residual correlations were close to the critical value for some of these item pairs, i.e., between the items about breathlessness in the ASTA symptom scale and between the items about *deteriorated life situation* and *carry out daily activities* in the ASTA HRQoL scale. Further, the findings about local dependency for the symptoms *weakness/fatigue vs. tiredness* and *spending less time with family/relatives and friends vs. spending less time with acquaintances* are not surprising. During the development phase of the ASTA questionnaire, patients suggested to have these questions separated with the explanation that it differed to be with persons you are familiar with or being with acquaintances and that *weakness/fatigue* is not the same as *tiredness* [4]. Criticism of local independence shows that it is difficult for persons’ responses to be completely independent of each other [24].

Despite some problems with local dependency, the combined principal component analysis of residuals and t-test approach supported unidimensionality for both the ASTA symptom and HRQoL scales. The validation of the original Swedish version of the ASTA questionnaire was based on Classic Test Theory (CTT), and confirmatory factor analysis (CFA) was used to evaluate the hypothesized factor structure and revealed significant factor loadings for the ASTA HRQoL 1-factor model, i.e., the 13-item total scale, with a partly unsatisfactory model fit. The CFA revealed high factor loadings in both models and it was found that the two-factor model had a better model fit [5]. The analysis led to a recommendation to use both the HRQoL total scale and the two subscales in the calculation of scores.

The person-item distributions showed that the ASTA questionnaire captures different levels of symptom burden and HRQoL, and the targeting was best for patients with a high symptom burden and poor HRQoL. This is advantageous for a clinical scale like the ASTA questionnaire; it implies that the patients with the most problems will be assessed. In order to improve targeting, additional items reflecting lower levels of symptom burden and higher levels of HRQoL may be considered.

The ASTA symptom and HRQoL scales demonstrated satisfactory reliability according to the person separation index and Cronbach´s alpha. This is in line with the validation studies of the Swedish version of the ASTA questionnaire where both scales had Cronbach’s alpha values above ≥ 0.7 [4, 5].

DIF is an often-overlooked aspect of validity and it is a strength to find that the ASTA questionnaire did not explore any DIF regarding gender or age. Valid comparisons need items to work invariantly between groups [25]. Thus, these findings indicate that the ASTA symptom and HRQoL scales can be used as invariant measures to make valid comparisons between groups of different sex and age. However, due to the restricted sample size, presence of type I errors can not be excluded. Thus, DIF should be further investigated in future evaluations of the ASTA questionnaire.

### Methodological considerations/limitations

This is a single center study, but includes a sufficient number of patients for Rasch analysis. The sampling procedure may have contributed to sampling bias. However, psychometric studies are commonly based on non-probability sampling since precisely accurate statistics may not be needed [26]. The patients were asked to comment on whether they had any uncertainties regarding understandability and the wording but no one pointed anything out concerning this. The most common issue the patients encountered was about their medications. They found this section difficult as they could not always remember or they did not know what they were on. In patients less symptomatic, questions regarding frequency and duration of AF episodes rendered in some perplexities. In the future, for further validation, cognitive debriefing or cognitive interviews can be considered.

There is no data available regarding Rasch analysis for the Swedish version of the ASTA questionnaire, and comparisons with the validation work on the original ASTA questionnaire are difficult because the original validation was carried out using CTT.

## Conclusions

The English version of the ASTA questionnaire demonstrated satisfactory measurement properties according to the Rasch model. However, it needs to be evaluated in a larger sample, preferably including patients with other forms of arrhythmia. Most important, the response categories in the English version should be considered in the ASTA questionnaire as well as DIF in further validation. The English version of the ASTA questionnaire can be used for assessments of symptoms and HRQoL between groups of different age and gender in patients with arrhythmia.

## Data Availability

The datasets used and/or analyzed during the current study are available from the corresponding author on reasonable request.

## Abbreviations

AF: Atrial fibrillation
AFL: Atrial flutter
ASTA: Arrhythmia-Specific questionnaire in Tachycardia and Arrhythmia
CFA: Confirmatory factor analysis
CTT: Classic test theory
DIF: Differential item functioning
ESC: European society of cardiology
HRQoL: Health-related quality of life
NOACs: New oral anti-coagulants
ICF: International classification of functioning
ISPOR: Professional Society for Health Economics and Outcomes Research
PROMs: Patient-reported outcome measures
PCA: Principal component analysis
SCL: Symptom checklist frequency and severity scale
